# Parental occupational exposure to pesticides and risk of childhood cancer in Switzerland: a census-based cohort study

**DOI:** 10.1186/s12885-020-07319-w

**Published:** 2020-08-28

**Authors:** Astrid Coste, Helen D. Bailey, Mutlu Kartal-Kaess, Raffaele Renella, Aurélie Berthet, Ben D. Spycher

**Affiliations:** 1 Institute of Social and Preventive Medicine, University of Bern, Bern, Switzerland; 2 Telethon Kids Institute, University of Western Australia, Perth, Western Australia Australia; 3 Division of Paediatric Haematology and Oncology, Department of Paediatrics, Inselspital, University of Bern, Bern, Switzerland; 4 Pediatric Haematology-Oncology Unit, Division of Pediatrics, Centre Hospitalier Universitaire Vaudois, Lausanne, Switzerland; 5 Center for Primary Care and Public Health (Unisanté), University of Lausanne, Lausanne, Switzerland

**Keywords:** Childhood cancer, Pesticides, Occupation, Record-based cohort

## Abstract

**Background:**

Pesticide exposure is a suspected risk factor for childhood cancer. We investigated the risk of developing childhood cancer in relation to parental occupational exposure to pesticides in Switzerland for the period 1990–2015.

**Methods:**

From a nationwide census-based cohort study in Switzerland, we included children aged < 16 years at national censuses of 1990 and 2000 and followed them until 2015. We extracted parental occupations reported at the census closest to the birth year of the child and estimated exposure to pesticides using a job exposure matrix. Cox proportional hazards models, adjusted for potential confounders, were fitted for the following outcomes: any cancer, leukaemia, central nervous system tumours (CNST), lymphoma, non-CNS solid tumours.

**Results:**

Analyses of maternal (paternal) exposure were based on approximately 15.9 (15.1) million-person years at risk and included 1891 (1808) cases of cancer, of which 532 (503) were leukaemia, 348 (337) lymphomas, 423 (399) CNST, and 588 (569) non-CNS solid tumours. The prevalence of high likelihood of exposure was 2.9% for mothers and 6.7% for fathers. No evidence of an association was found with maternal or paternal exposure for any of the outcomes, except for “non-CNS solid tumours” (High versus None; Father: adjusted HR [95%CI] =1.84 [1.31–2.58]; Mother: 1.79 [1.13–2.84]). No evidence of an association was found for main subtypes of leukaemia and lymphoma. A post-hoc analysis on frequent subtypes of “non-CNS solid tumours” showed positive associations with wide CIs for some cancers.

**Conclusion:**

Our study suggests an increased risk for solid tumours other than in the CNS among children whose parents were occupationally exposed to pesticides; however, the small numbers of cases limited a closer investigation of cancer subtypes. Better exposure assessment and pooled studies are needed to further explore a possible link between specific childhood cancers types and parental occupational exposure to pesticides.

## Background

The causes of childhood cancers are still largely unknown. Although rare, they constitute the most common disease-related cause of death among children in many high income countries including Switzerland [1]. The most common cancer types in childhood are leukaemia, tumours of the central nervous system (CNST) and lymphoma [1]. Moderate to high doses of ionising radiation are known to cause leukaemia and CNST [2, 3]. Furthermore, certain genetic disorders, including DNA repair disorders, as well as exposure to chemotherapy are known to increase the risk of certain types of childhood cancers [2, 3]. Numerous environmental risk factors have been suspected to contribute to the risk of childhood cancer including exposure to pesticides [2, 4]. Pesticides cover a wide range of substances and active ingredients. There is evidence of carcinogenic effects for some pesticides from animal experiments, mechanistic studies and epidemiological studies of occupationally exposed adults [5]. Certain pesticides have been classified as carcinogenic to human by the International Agency for Research on Cancer (IARC) [6–8].

Children may have lower levels of exposure to pesticides than occupationally exposed adults, but physiologic and behavioural characteristics may make them more vulnerable [9, 10]. The prenatal and early childhood periods are critical time windows of heightened susceptibility to environmental exposures [9]. Parental occupational exposure to pesticides may affect the child before conception, in utero and postnatally [11, 12]. Before conception, parental exposure may affect germ cells and during pregnancy, maternal exposure can result in foetal exposure [9, 12]. Children may ingest or inhale pesticide residues contained in dust, in the air or in the clothes of the occupationally exposed parents [10–13]. The ingestion of dust is particularly common in toddlers who still crawl and put objects into their mouths [10, 11].

Several epidemiological studies have investigated possible associations between parental occupational exposure and cancer risks in children. For childhood leukaemia, two systematic reviews and meta-analyses of such studies concluded that there was evidence of a positive association with leukaemia [14, 15]. A large study from the Childhood Leukemia International Consortium (CLIC) using pooled data from 13 case-control studies and a harmonized job exposure matrix (JEM) to assess exposure showed a positive association between occupational maternal exposure to pesticides during pregnancy and the risk of acute myeloid leukaemia (AML). It was also suggestive of an association between paternal exposure around conception and acute lymphoblastic leukaemia (ALL) [16]. For CNST, a review and meta-analysis of 20 studies suggested a positive association with parental occupational exposure to pesticides [17]. A prospective study from the International Childhood Cancer Cohort Consortium (I4C) pooling data from five birth cohorts reported an increased risk for AML but not for ALL or CNST in the offspring of occupationally exposed fathers [18]. There have been fewer studies on childhood lymphoma and their findings are inconsistent [19–22]. Most previous studies on childhood cancers were interview-based case-controls studies that may be subject to recall and selection bias. The possible link between parental occupational exposure to pesticides and childhood cancer warrants further investigation in other settings, with rarer cancers, and with study designs that minimise the risk of bias.

In this study, we investigated the risk of childhood cancer and its main diagnostic groups following parental occupational exposure to pesticides in a nationwide census-based cohort in Switzerland. Exposure assessment was based on self-reported occupations at censuses and a JEM developed for the previous pooled case-control study of the CLIC consortium [16].

## Methods

### Population data

This study was based on the childhood population in the Swiss National Cohort (SNC) study during the period 1990 to 2015. The SNC is a linkage-based cohort including all individuals recorded in the decennial censuses 1990, 2000 and annual registry-based censuses from 2010 onward. Censuses collected data on socio-demographic characteristics including current occupation. Probabilistic record linkage was used to link individual records across censuses and with records from national datasets on mortality, live births and emigration [23, 24].

We included all children aged 0–15 years old at census 1990 or census 2000 for whom at least one parent could be identified. The census questionnaire did not permit direct identification of biological parents, so parents were identified by attributing adults reporting to have children to matching children living in the same household. Children were followed from the date of the first census they were recorded in (entry time point) until first occurrence of one of the following events: 16^th^ birthday, death, migration, lost to follow-up, administrative censoring (31/12/2015) or cancer diagnosis.

### Case ascertainment

We identified primary diagnoses of cancers among eligible children through probabilistic record linkage with the Swiss Childhood Cancer Registry (SCCR). The SCCR is a population-based registry with nationwide coverage for children aged 0–15 years and high completeness (≥ 95% for the period 1995–2009) [25]. We used the following variables to match cancer diagnosis with SNC records: sex, date of birth, parental dates of birth, geocoded residence at census, first names (available only for children born in Switzerland), municipality of residence at census and at birth and nationality. We excluded children with a cancer diagnosis occurring before entry into the cohort.

### Outcomes

Cancer diagnoses were coded using the International Classification of Childhood Cancer (ICCC-3) [26]. We separately investigated following outcomes: Any cancer (all cancers or all ICCC-3 diagnostic groups); leukaemia (ICCC-3 main diagnostic group I); lymphoid leukaemia (LL) (ICCC-3 diagnostic group I a); acute myeloid leukaemia (AML) (ICCC-3 diagnostic group I b); lymphoma (ICCC-3 main diagnostic group II); Non-Hodgkin lymphoma (NHL) (ICCC-3 diagnostic group II b and c); Hodgkin lymphoma (ICCC-3 diagnostic group II a); CNST (ICCC-3 main diagnostic group III; this also includes tumours of non-malignant or uncertain behaviour) and non-CNS solid tumours (ICCC-3 main diagnostic groups IV to XII).

### Exposure assessment

Parental occupation was determined from the job title declared by parents at time of entry (first census) into the cohort. Job titles were assigned to four-digit codes of the International Standard Classification of Occupation (ISCO) 1988 as in an earlier study [27] and were linked to a JEM (referred to here as CLIC-JEM) previously developed for a pooled study by the Childhood Leukemia International Consortium (CLIC) [16]. The development of the CLIC-JEM is based on data from an Australian study in CLIC and expert assessment of pesticide exposure and is described elsewhere [28]. Briefly, for each job code in the ISCO 08 system, the proportion of jobs assessed as involving exposure to pesticides was calculated [28]. Based on this, the likelihood of exposure was classified into 4 categories: 1) ‘High likelihood of pesticide exposure’: ≥70% of people with these ISCO-08 codes exposed to pesticides; 2) ‘Moderate likelihood of exposure’: 25–70% exposed; 3) ‘Limited likelihood of exposure’: 10–25% exposed 4) ‘No or minimal likelihood of pesticide exposure’: < 10% exposed [28]. Further refinements of the JEM were made using data from a Canadian study in CLIC and similar methods [29]. The final exposure codes in the JEM were then assigned to equivalent ISCO-88 codes. Given of the high uncertainty about the probability of exposure in categories 2 and 3, the JEM was intended to be used only to compare those with a high likelihood of exposure to those with no or minimal likelihood of exposure. The final list of ISCO-88 job titles in the high likelihood category are listed in S1.

Some job titles reported by parents in the SNC only had three-digit ISCO codes and could not be assigned a unique exposure category using the JEM. For these codes, experts of occupational exposure assessment (Lin Fritschi, who participated in the development of CLIC-JEM and co-authors HB and AB) assessed the likelihood of exposure based on the original job title text reported by the parents. Children of parents who did not report a job title and where coded as not economically active (unemployed, still in education, housework in own home, retired, other unpaid work) were classified as having “no or minimal parental exposure”. We excluded children of parents whose reported job title could not be assigned a likelihood of exposure by the experts.

### Potential confounders

As potential confounders we considered covariates that were found to be associated with childhood cancer in previous studies based on the SNC and/or suspected risk indicators for which data was available. To guide our selection, we constructed a directed acyclic graph (S2). As a result, we included the following factors: education of the reference person in the household (compulsory or less, secondary level, tertiary level); maternal age at birth (< 25 years old, 25–29, 30–34, ≥35) [30]; parental occupational exposure to benzene based on a previously used JEM (4 exposure categories combining probability and level of exposure) [27]; the Swiss neighbourhood index of socioeconomic position (Swiss-SEP) (quintiles) [31]; modelled air concentration of NO_2_ (pg/m^3^) as a surrogate of air pollution (as continuous variable); modelled dose rate of ionising background radiation including terrestrial gamma and cosmic radiation (< 100, 100–150, 150–200, ≥250 nSv/h) [32].

### Statistical analysis

We used Cox proportional hazards regression models with age as the underlying time scale to estimate hazard ratios (HRs) and 95% confidence intervals (CIs) comparing the risk of childhood cancer across different exposure categories. We ran separate analyses for maternal exposure including all eligible mother-child pairs and paternal exposure including all eligible father-child pairs. In our main analysis, hazard ratios with 95% CIs are reported comparing “high likelihood of exposure” to “no or minimal exposure”. Results from “moderate” and “limited” likelihood of exposure are only shown in supplementary material, due to the uncertainty of exposure level in these categories. We included potential confounders in two steps. The initial model was adjusted only for sex, birth year and entry year (either 1990 or 2000). We regarded the entry year as potential effect modifier because the proportion of people exposed markedly decreased between these two censuses time points. We used likelihood ratio tests to test for interaction between entry year and exposure. In a second step, we included all potential confounders listed above.

In sensitivity analysis, we excluded parent-child pairs with non-economically active parents (unemployed, still in education, housework in own home, retired, other unpaid work). In the SNC, many mothers reported occupations categorized as “housework in own home/homemaker”. In a second sensitivity analysis, we assumed that a mother also had a high likelihood of exposure if the child’s father had an occupation in agriculture (ISCO-codes 6111, 6112, 6113, 6121, 6130 and 9211). In additional analyses, we also investigated exposure of either parent (at least one with high likelihood of exposure) versus no parents exposed.

## Results

**Fig. 1:**
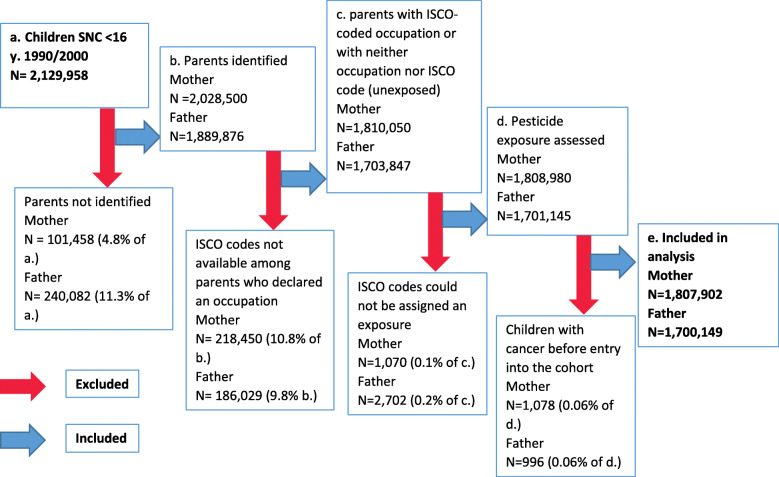
Flow chart of children included in the main analysis

We identified mothers of 95% of the children aged 0–15 years in the SNC, of whom 56% reported an occupation when their children entered the cohort (Fig. 1). While fewer fathers were identified (89%), a higher proportion reported an occupation (88% of those identified). Among these declared occupations, around 10% did not have an ISCO code (Fig. 1). More than 99% of the available ISCO codes could be linked with the CLIC-JEM and assigned an exposure. We also included parents not economically active and without a declared occupation as non-exposed. After excluding children who developed cancer before entry into the cohort, our final analysis included 1,807,902 mother-child and 1,700,149 father-child pairs, with 1,407,503 children included in both groups (Fig. 1). While the characteristics of children included for the analyses of maternal and paternal exposure were similar, children excluded from the main analysis tended to have a reference person in the household with comparatively lower level of education and were more likely to live in a deprived or urban area (Table 1).

**Table 1:** Characteristics at the time of entry into the Swiss National Cohort of the children included and excluded from analyses

Characteristics	Maternal exposure		Paternal exposure		Excluded from analysis^**1**^	
	n	%	n	%	n	%
**Total**	1,807,902	*100*	1,700,149	*100*	172,974	*100*
**Sex**						
Female	882,885	*48.8*	829,089	*48.8*	82,926	*47.9*
**Children age at entry (years)**						
0–4	681,683	*37.7*	652,023	*38.4*	58,498	*33.8*
5–9	671,441	*37.1*	628,993	*37.0*	73,083	*42.3*
10–14	454,778	*25.2*	419,133	*24.7*	41,393	*23.9*
**Education of reference person**^**2**^ **in the household**						
Compulsory education or less	315,095	*17.4*	275,748	*16.2*	42,878	*24.8*
Upper secondary level education	929,231	*51.4*	873,756	*51.4*	50,403	*29.1*
Tertiary level education	543,703	*30.1*	538,377	*31.7*	23,921	*13.8*
Not known	19,873	*1.1*	12,268	*0.7*	55,772	*32.2*
**Swiss-SEP**^**3**^						
Q1	480,442	*26.6*	447,167	*26.3*	54,900	*31.7*
Q2	371,151	*20.5*	349,556	*20.6*	34,861	*20.2*
Q3	343,012	*19.0*	323,173	*19.0*	28,202	*16.3*
Q4	322,421	*17.8*	304,985	*17.9*	22,717	*13.1*
Q5	275,612	*15.2*	262,061	*15.4*	16,620	*9.6*
missing	15,264	*0.8*	13,207	*0.8*	15,674	*9.1*
**Degree of urbanization**						
Urban	422,132	*23.3*	375,466	*22.1*	55,893	*32.3*
Semi-urban	834,780	*46.2*	784,674	*46.2*	72,060	*41.7*
Rural	550,990	*30.5*	540,009	*31.8*	45,021	*26.0*
**Parents’ occupational exposure to pesticides**						
High likelihood (≥70% of people exposed)	53,074	*2.9*	113,784	*6.7*	NA	
Moderate likelihood (≥25 to 70%)	1233	*0.1*	30,717	*1.8*		
Limited likelihood (≥10 to 25%)	31,838	*1.8*	104,304	*6.1*		
No or minimal likelihood (< 10%)	1,721,757	*95.2*	1,451,344	*85.4*		

Data represent number of children and column percentages *(in italic).* NA: data not available. *P*-values of chi-squared tests for differences between included and excluded children were < 0.001 for all socio-demographic characteristics)

^1^Children were excluded from both analyses if neither their father nor mother could be identified or could not be assigned an exposure due to missing or non-classifiable job titles (Fig. 1)

^2^Person that contributes the most to the income of the household

^3^The SEP-index is an area-based measure of socio-economic position for Switzerland, estimated in neighbourhoods of 50 households with a principal component analysis of four socio-economic variables, with data from census 2000^26^

Only a small minority of fathers (6.7%) and mothers (2.9%) of included children were classified as highly likely to be exposed to pesticides, while the vast majority of fathers (85.4%) and mothers (95.2%) had minimal likelihood of exposure (Table 1). The proportion of people with high likelihood of exposure decreased between 1990 and 2000 (7.1 vs. 5.8% for fathers; 3.7 vs. 1.5% for mothers, S3). Among parents with high likelihood of exposure to pesticides, the most prevalent ISCO-88 job category was “Market-oriented crop and animal producers” (S1) (prevalence around 80%). Among children with mothers who were highly likely to be occupationally exposed, 87% also had a father who had high likelihood of exposure (S4).

Parents highly likely to be exposed to pesticides tended to have a lower education level, to live in a rural area and/or a more deprived neighbourhood, to have a lower exposure level to background radiation and air pollution than parents with a minimal likelihood of pesticide exposure (S5 and S6). Parents with high likelihood of occupational pesticide exposure were not classified as occupationally exposed to benzene according to a previously used JEM (S5 and S6) [27].

Among children included for analysis of paternal exposure we identified 1808 incident cases of childhood cancer including 503 (27.8%) with leukaemia, 337 (18.6%) with lymphoma, 399 (22.1%) with CNST, and 569 (31.5%) with non-CNS solid tumours (S7). For the mother-child pairs, we identified 1891 childhood cancers, with a similar distribution of diagnostic groups: 532 (28.1%) leukaemia, 348 (18.4%) lymphoma, 423 (22.4%) CNST and 588 (31.1%) non-CNS solid tumours (S7). The proportions of parents exposed were similar among cancer cases (S7). For some diagnostic groups such as AML, NHL and HL, there were < 10 cases in the highest exposure category even for paternal exposure (S7).

We found no evidence for an association between a high likelihood of paternal pesticide exposure and the risk of any cancer (all cancers combined) in the offspring. Adjusting for potential confounders, the HR comparing high likelihood of exposure to no or minimal likelihood of exposure was 1.14 [95% CI: 0.91–1.43] (Table 2, S8). Analysis by main diagnostic groups showed no evidence of an association between paternal exposure and the risk of leukaemia, lymphoma or CNST (Table 2, S8). There was, however, evidence of an increased risk for “non-CNS solid tumours” for the highest exposure category: fully adjusted HR 1.84 [1.31–2.58] (Table 2, S8). Except for CNST, adjusting for potential confounding factors tended to increase HRs. We found no evidence of an association with paternal exposure for LL, AML, NHL or HL (Table 3).

**Table 2:** Association between parental occupational exposure to pesticides and risk of childhood cancer in the Swiss National Cohort; major diagnostic groups only

		Paternal exposure			Maternal exposure		
Outcome	Likelihood of Exposure	Cases	Partially adjusted model^1^	Fully adjusted model^2^	Cases	Partially adjusted model^1^	Fully adjusted model^2^
		n	HR [95%CI]	HR [95%CI]	n	HR [95%CI]	HR [95%CI]
Any cancer	Minimal	1559	1	1	1808	1	1
	High	112	0.95 [0.79–1.16]	1.14 [0.91–1.43]	49	1.00 [0.75–1.33]	1.13 [0.82–1.56]
Leukaemia	Minimal	438	1	1	515	1	1
	High	24	0.73 [0.49–1.10]	0.79 [0.48–1.29]	9	0.66 [0.34–1.27]	0.66 [0.29–1.49]
Lymphoma	Minimal	297	1	1	333	1	1
	High	21	0.92 [0.59–1.43]	1.06 [0.63–1.78]	9	0.96 [0.49–1.86]	1.18 [0.57–2.44]
CNST	Minimal	345	1	1	405	1	1
	High	20	0.78 [0.50–1.22]	0.76 [0.44–1.33]	8	0.77 [0.38–1.55]	0.65 [0.26–1.60]
Non-CNS solid tumours	Minimal	479	1	1	555	1	1
	High	47	1.30 [0.96–1.75]	1.84 [1.31–2.58]	23	1.49 [0.98–2.26]	1.79 [1.13–2.84]

CNST: central nervous system tumour; HR: Hazard Ratio estimated with a Cox regression; 95%CI: 95% confidence interval

^1^Model adjusted for sex, birth year and year of entry

^2^Model adjusted for sex, birth year, year of entry, maternal age at birth, paternal and maternal occupational exposure to benzene, education level of the reference person in the household, SEP-index, degree of urbanization, residential exposure to background ionizing radiation, residential exposure to ambient NO_2_ (All variables assessed at entry into the cohort)

**Table 3:** Association between parental occupational exposure to pesticides and risk of childhood cancer in the Swiss National Cohort; leukaemia and lymphoma subtypes

		Paternal exposure			Maternal exposure		
Outcome	Likelihood of Exposure	Cases	Partially adjusted model^1^	Fully adjusted model^2^	Cases	Partially adjusted model^1^	Fully adjusted model^2^
		n	HR [95%CI]	HR [95%CI]	n	HR [95%CI]	HR [95%CI]
LL	Minimal	332	1	1	397	1	1
	High	19	0.77 [0.48–1.22]	0.73 [0.41–1.31]	8	0.76 [0.38–1.54]	0.69 [0.28–1.71]
AML	Minimal	75	1	1	84	1	1
	High	3	0.53 [0.17–1.68]	0.97 [0.29–3.25]	1	0.42 [0.06–3.04]	0.86 [0.11–6.50]
NHL	Minimal	151	1	1	166	1	1
	High	8	0.69 [0.34–1.41]	0.75 [0.32–1.75]	4	0.84 [0.31–2.27]	1.16 [0.41–3.23]
HL	Minimal	140	1	1	162	1	1
	High	13	1.21 [0.68–2.13]	1.43 [0.74–2.77]	4	0.89 [0.33–2.41]	1.27 [0.45–3.56]

LL: lymphoid leukaemia; AML: acute myeloid leukaemia; NHL: Non-Hodgkin lymphoma; HL: Hodgkin lymphoma; HR: Hazard Ratio estimated with a Cox regression; 95%CI: 95% confidence interval

^1^Model adjusted for sex, birth year and census year at entry

^2^Model adjusted for sex, birth year, census year at entry, maternal age at birth, paternal and maternal occupational exposure to benzene, education level of the reference person in the household, SEP-index, degree of urbanization, residential exposure to background ionizing radiation, residential exposure to ambient NO_2_ (All variables assessed at entry into the cohort)

Results for maternal exposure showed a closely similar pattern to those for paternal exposure (Table 2, S8). No evidence of an association was found for any cancer (all cancers combined), leukaemia, lymphoma, NHL or CNST (Table 2, S8), or for the subtypes of leukaemia and lymphoma (Table 3). However, there was evidence of an increased risk of “non-CNS solid tumours” among children of mothers highly likely to be exposed (fully adjusted HR 1.79, 95% CI 1.13–2.84).

We observed weak evidence that exposure outcome associations differed between children who entered the cohort in 1990 and those who entered in 2000 for any cancer (*P*-value of interaction test =0.02) and lymphoma (*P* = 0.03) with paternal exposure; and for any cancer with maternal exposure (*P* = 0.04). For these outcomes, a stratified analysis suggested negative associations for children entering in 1990 and positive associations for children entering in 2000 (S9). For the other diagnostic groups, we found no evidence of interaction (*P* > 0.05).

Results remained virtually unchanged in the sensitivity analysis considering non-economically active parents as missing (S10). Similarly, reclassifying mothers reporting housework as probably exposed if the father reported an occupation in agriculture (ISCO-codes 6111, 6112, 6113, 6121, 6130 and 9211, see S4) had little impact on the results (S11). Results were similar in the separate analyses comparing children with at least one parent having high likelihood of exposure to children whose parents both had minimal likelihood of exposure (S12).

Given the evidence of association with paternal and maternal exposure for the group of non-CNS solid tumours, we conducted exploratory post-hoc analyses for some frequent subtypes in this group. We investigated subgroups with at least 50 cases in the samples for maternal or paternal exposure. These analyses were suggestive of increased risks for “malignant bone tumours” (mothers and fathers) and “soft tissue and other extraosseous sarcomas” (mothers and fathers) among children whose parents had high likelihood of exposure **(**Table 4**)**. However, CIs were wide and compatible with negative associations. The lower bound of the 95%-CI was highest for malignant bone tumours and maternal exposure (1.95, 95% CI: 0.91, 4.18) (Table 4).

**Table 4:** Post-Hoc analysis on most frequent^1^ subgroups in the “other cancer” category

		Paternal exposure		Maternal exposure	
Outcome	Likelihood of Exposure	Cases	Partially adjusted model^2^	Cases	Partially adjusted model^2^
		n	HR [95%CI]	n	HR [95%CI]
Renal tumours	Minimal	47	1	54	1
	High	4	1.18 [0.42–3.27]	3	2.19 [0.68–7.05]
Malignant bone tumours	Minimal	105	1	125	1
	High	12	1.49 [0.82–2.71]	7	1.95 [0.91–4.18]
Soft tissue and other extraosseous sarcomas	Minimal	99	1	123	1
	High	12	1.61 [0.88–2.93]	6	1.79 [0.79–4.1]
Other malignant epithelial neoplasms and malignant melanoma	Minimal	79	1	87	1
	High	6	1.01 [0.44–2.32]	2	0.86 [0.21–3.52]
Germ cell tumours, trophoblastic tumours, and neoplasms of gonads	Minimal	45	1	52	1
	High	5	1.45 [0.58–3.67]	2	1.43 [0.35–5.91]

HR: Hazard Ratio estimated with a Cox regression; 95%CI: 95% confidence interval

^1^Subgroups with sum of cases with minimal and high likelihood of exposure ≥50

^2^Model adjusted for sex, birth year and census year at entry

## Discussion

This nationwide census-based cohort study found no evidence of an association between the risk for main diagnostic groups of childhood cancer and parental occupational exposure to pesticides, neither for maternal nor for paternal exposure. However, the study did show evidence of an increased risk for the heterogeneous group “non-CNS solid tumours” among children with high likelihood of maternal and paternal occupational exposure to pesticides. An exploratory post-hoc analysis on the frequent non-CNS solid tumours showed a positive association with bone tumours and soft tissue and other extraosseous sarcomas, but with wide CIs. Adjustment for potential confounders and sensitivity analyses with modifications in exposure classification showed similar results.

In contrast to our study, a large international analysis based on the same JEM and pooled data from 13 case-control studies of the CLIC consortium [16], and previous meta-analyses [14, 15, 33] reported a positive association between childhood leukaemia and parental occupational pesticide exposure. In the literature, the highest and most consistent effects [14, 16] were observed between risk of AML and maternal occupational exposure to pesticides during pregnancy. Furthermore, prenatal exposure to certain insecticides has been associated with translocations found in children with AML [34, 35]. Studies of domestic use of pesticides have shown a consistent association between such pesticide use during pregnancy and risk of childhood AML [33, 36]. Regarding paternal occupational exposure, the CLIC study found a slight increase of risk of ALL, for exposure around conception [16]. The I4C cohort, a recent study of pooling data from 5 birth cohorts found no association for ALL, but an increased risk of AML in offspring of fathers exposed to pesticides during pregnancy [18]. The differences observed in our study compared to others might be due to the small sample sizes and lack of statistical power.

For CNST, our study is consistent with both a recent pooled case-control study [37] and the international I4C pooled birth cohort study [18], neither of which found evidence of an association. As in our study, these previous studies had a limited sample size. In contrast, a meta-analysis focusing only on childhood brain tumours (a subgroup of CNST) found an increased risk in offspring of parents occupationally exposed to pesticides, especially of mothers exposed during pregnancy [17], while another meta-analysis reported a positive association with paternal exposure, most pronounced for postnatal exposure [33].

As in our study, no evidence of an association for lymphoma was found in a recent large record-based British case-control study using paternal occupation recorded in the birth registration [21]. However, an earlier meta-analysis did suggest that parental domestic use of pesticides was associated with a higher risk of childhood lymphoma [33]. Although some case-control and cohort studies suggested an association with parental occupational exposure, these were based on small number of cases [19, 20, 22].

No other study has separately investigated the aggregated group “non-CNS solid tumours”. Our post-hoc analysis on subtypes of the group suggested positive associations with malignant bone tumours and soft tissue sarcomas for both paternal and maternal exposure, but all estimates lacked precision. There have been few reports about these rare cancers. A meta-analysis showed positive associations between paternal occupational exposure to pesticides and Ewing’s sarcomas, a bone tumour [33]. However, these results were based on small numbers of cases. Similarly, a review on the epidemiology of bone tumours which included more studies concluded that parental occupation as a farmer was consistently associated with all bone tumours [38].

Discrepancies between findings across studies may be also related to study design. A previous meta-analysis [14] on leukaemia and parental occupational exposure to pesticides noted that case-control studies tended to find higher estimates compared to cohort studies. Case-control studies were often interview-based and may have been susceptible to selection and recall biases. However, positive associations with leukaemia were also found in the recent meta- and pooled analysis [16] by the CLIC consortium, which used a JEM to assess exposure that would have limited the risk of recall bias. Also, cohort studies have often been underpowered and may thus have failed to detect a potential effect.

By including data from nationwide registration over a period of 26 years, our study included a relatively high numbers of cases for the main groups of childhood cancers (leukaemia, lymphoma, CNST) compared to some previous cohort studies. Numbers of exposed cases were however small, particularly for diagnostic subgroups and maternal exposure, which was rarer. The SCCR is a population-based cancer registry of high coverage. However, linkage errors or incomplete linkage (some SCCR cases could not be linked) may have resulted in some misclassification of outcomes.

Our study was based on occupations reported during compulsory national censuses, which minimizes the risk of differential misclassification of exposure. Unlike most of previous studies which were case-control studies, this study assessed exposure before diagnosis of cancer in children, so was not prone to recall bias. However, we cannot rule out the possibility of selection bias, as a considerable proportion of children had to be excluded because their parents could not be identified or could not be assigned an exposure for other reasons. These children tended to have a lower socio-economic status and to live more frequently in urban area compared to the included children. Although we were able to adjust analyses for a number of area-based socio-economic and environmental factors, information on individual behavioural factors such as parental smoking status or exposure to infections was not available. Therefore, we cannot exclude residual confounding by unobserved factors. We also do not have information on other sources of pesticide exposure, such as domestic use of pesticides by parents inside homes and in gardens, or such as exposure through drift of pesticides spread in crop fields near the residence. It is likely that parental occupational exposure to pesticides is highly correlated with high levels exposure to pesticide drift from crop fields. The inability to adjust for these other sources may have resulted in point estimates being closer to the null.

A major limitation of our study was that child’s age at the time of exposure assessment was determined by the census date, and thus uniformly spread over the ages 0–15 years old. We had no information on exposure before birth, which may be a critical time for pesticides exposure. However, most of the studies that had more precise time windows of exposure [14, 16] found high correlation between prenatal and early childhood exposure. In our cohort, among parents with a reported occupation in 1990 and 2000, we observed that 90.4% of fathers with “high likelihood of exposure to pesticides” in 1990 were still in the same category of exposure in 2000; while for mothers this percentage was 70.4% (S13). Among parents who were not exposed in 1990, more than 95% (mothers and fathers) were still not exposed in 2000 (S13). Thus, in this sample, exposure of mothers at their child’s entry into the cohort might not accurately reflect their exposure at other time windows. Furthermore, a job title may not reflect the actual tasks performed. For example, during pregnancy women often modify their tasks which may not be detected even in studies with time-specific data. The use of a JEM to assess exposures may have resulted in considerable (non-differential) exposure misclassification potentially diluting any existing associations with outcomes. The JEM was developed based on expert assessments of occupational exposures in Australia and Canada [16]. Application to the Swiss setting, where exposure patterns in occupational categories likely differ from these countries [39], may have increased the potential for misclassification. Pesticides comprise numerous active ingredients, only some of which may be carcinogens, and use of these might considerably vary between countries [39]. Furthermore, the JEM used here only assesses probability of exposure without taking into account exposure levels and protective measures. In a Californian study [40], the OR measuring the association between childhood ALL and paternal occupational exposure was attenuated by 57% when using this JEM compared to a more elaborate exposure assessment method. This demonstrates a real possibility of effect dilution.

Various reasons might explain why our study did not find support for the associations found in previous studies and meta-analysis between parental occupational exposure and risk of childhood leukaemia and CNST. First, our study may have been insufficiently powered. Second, farm structures and farming practices and the balance between pesticide exposure and other exposures may differ in Switzerland from other countries. Swiss farms are smaller on average (mean = 17.8 ha in 2010) [41] compared to France (mean = 56 ha in 2010) [42] or Australia (mean = 4331 ha in 2015–2016) [43] according to national Farm censuses. In particular Switzerland has one of the highest livestock densities in Europe, with around 1.71 livestock units per hectares of utilized agricultural area in 2010 [44]. Previous studies have found a reduced risk of childhood ALL and lymphoma among children with early life exposure to farm animals [45–47], while increased risks were found for other cancer subtypes, such as AML, CNST, germ-cell tumours and astrocytoma [18, 47]. Given that about 80% of parents with high likelihood of exposure category belonged to job category “market-oriented crop and animal producers” (S1), it is possible that our analysis was confounded by exposure to farm animals.

The positive associations observed in our study for the heterogeneous group of “Non-CNS solid tumours” require further investigation. This is particularly true for our positive associations seen for malignant bone tumours and soft tissue sarcomas, which, though in line with some findings in the literature [38], are based on exploratory post-hoc analyses.

## Conclusion

Our study does not provide support of an association of leukaemia, CNST and lymphoma risk with postnatal parental exposure to pesticides. Our findings are suggestive of an association with non-CNS solid tumours, particularly for malignant bone tumours and soft tissue sarcomas. Further studies including detailed exposure assessments and links with data on occupational co-exposures could help to improve our understanding of the specific effects of parental occupational exposure to pesticides on childhood cancer risk. Pooled studies will also be necessary to investigate effects on rare cancer subtypes.

## Supplementary information

**Additional file 1 **S1: Job categories assigned a high likelihood of exposure to pesticides by CLIC-JEM and their prevalence among parents of children included in the study. S2: Directed Acyclic Graphs (DAG) of known and suspected associations with childhood cancers (Potential confounders for which data were available and which were considered in the analyses are shown in bold). S3: Exposure prevalence among parents of children included in the analysis based on CLIC-JEM and reported job categories at censuses 1990 and 2000. S4: Frequency of paternal and maternal occupational exposure to pesticides among children included in both analyses (*n* = 1,407,503). S5: Association between potential confounders and likelihood of paternal exposure to pesticides. S6: Association between potential confounders and likelihood of maternal exposure to pesticides. S7: Cases of childhood cancers included in analyses and likelihood of parental occupational exposure to pesticides. S8: Association between parental occupational exposure to pesticides and risk of childhood cancer in the Swiss National Cohort. S9: Association between parental occupational exposure to pesticides and risk of childhood cancer in the Swiss National Cohort stratified by census year of entry (only outcomes with evidence of interaction shown). S10: Association between parental occupational exposure to pesticides and risk of childhood cancer in the Swiss National Cohort, classifying non-economically active parents as missing. S11: Sensitivity analysis classifying mothers reporting “housework” as having high likelihood of exposure if the father reported an occupation in agriculture. S12: Additional analysis comparing children for whom at least one parent had high likelihood of exposure to children whose parents both had no or minimal exposure. S13: Change of parental occupational pesticides exposure between 1990 and 2000.

## Data Availability

The datasets generated and/or analysed during the current study are not publicly available due to local data protection policies. For further information about access to data from the Swiss National Cohort and the Childhood Cancer Registry please consult the respective websites: https://www.swissnationalcohort.ch/data-and-access/https://www.childhoodcancerregistry.ch/data/

## Abbreviations

SNC: Swiss National Cohort
SCCR: Swiss Childhood Cancer Registry
CLIC: Childhood Leukemia International Consortium
JEM: Job Exposure Matrix
IARC: International Agency for Research on Cancer
CLIC-JEM: Job Exposure Matrix built from the international pooled case-control study from the Childhood Leukemia International Consortium, conducted by IARC
ALOHA-JEM: A Job Exposure Matrix developed by the team of Dr. Roel Vermeulen from IRAS, Utrecht University
ISCO: International Standard Classification of Occupations
ICCC-3: International Classification of Childhood Cancer, Third edition
LL: Lymphoid Leukaemia according to ICCC-3
ALL: Acute Lymphoblastic Leukaemia (very close to LL group, but restriction on acute leukaemia)
AML: Acute Myeloid Leukaemia
CNST: Central Nervous System Tumour
NHL: Non-Hodgkin lymphoma
HL: Hodgkin lymphoma
CI: Confidence Intervals
HR: Hazards Ratio
