# Supplementary material for: Parental occupational exposure to pesticides and risk of childhood cancer in Switzerland: a census-based cohort study
Source: BMC Cancer. 2020 Aug 28;20:819. doi: 10.1186/s12885-020-07319-w (PMC7456012; doi:10.1186/s12885-020-07319-w)
Supplement: Supplementary file 1 — Additional file 1 S1: Job categories assigned a high likelihood of exposure to pesticides by CLIC-JEM and their prevalence among parents of children included in the study. S2: Directed Acyclic Graphs (DAG) of known and suspected associations with childhood cancers (Potential confounders for which data were available and which were considered in the analyses are shown in bold). S3: Exposure prevalence among parents of children included in the analysis based on CLIC-JEM and reported job categories at censuses 1990 and 2000. S4: Frequency of paternal and maternal occupational exposure to pesticides among children included in both analyses (n = 1,407,503). S5: Association between potential confounders and likelihood of paternal exposure to pesticides. S6: Association between potential confounders and likelihood of maternal exposure to pesticides. S7: Cases of childhood cancers included in analyses and likelihood of parental occupational exposure to pesticides. S8: Association between parental occupational exposure to pesticides and risk of childhood cancer in the Swiss National Cohort. S9: Association between parental occupational exposure to pesticides and risk of childhood cancer in the Swiss National Cohort stratified by census year of entry (only outcomes with evidence of interaction shown). S10: Association between parental occupational exposure to pesticides and risk of childhood cancer in the Swiss National Cohort, classifying non-economically active parents as missing. S11: Sensitivity analysis classifying mothers reporting “housework” as having high likelihood of exposure if the father reported an occupation in agriculture. S12: Additional analysis comparing children for whom at least one parent had high likelihood of exposure to children whose parents both had no or minimal exposure. S13: Change of parental occupational pesticides exposure between 1990 and 2000. [file 12885_2020_7319_MOESM1_ESM.docx]

# Supplementary material

## Table of contents

[S1: Job categories assigned a high likelihood of exposure to pesticides by CLIC-JEM and their prevalence among parents of children included in the study. 2](#_Toc48063697)

[S2: Directed Acyclic Graphs (DAG) of known and suspected associations with childhood cancers (Potential confounders for which data were available and which were considered in the analyses are shown in bold) 3](#_Toc48063698)

[S3: Exposure prevalence among parents of children included in the analysis based on CLIC-JEM and reported job categories at censuses 1990 and 2000 4](#_Toc48063699)

[S4: Frequency of paternal and maternal occupational exposure to pesticides among children included in both analyses (n= 1,407,503) 5](#_Toc48063700)

[S5: Association between potential confounders and likelihood of paternal exposure to pesticides 6](#_Toc48063701)

[S6: Association between potential confounders and likelihood of maternal exposure to pesticides 8](#_Toc48063702)

[S7: Cases of childhood cancers included in analyses and likelihood of parental occupational exposure to pesticides 10](#_Toc48063703)

[S8: Association between parental occupational exposure to pesticides and risk of childhood cancer in the Swiss National Cohort 11](#_Toc48063704)

[S9: Association between parental occupational exposure to pesticides and risk of childhood cancer in the Swiss National Cohort stratified by census year of entry (only outcomes with evidence of interaction shown) 12](#_Toc48063705)

[S10: Association between parental occupational exposure to pesticides and risk of childhood cancer in the Swiss National Cohort, classifying non-economically active parents as missing 13](#_Toc48063706)

[S11: Sensitivity analysis classifying mothers reporting “housework” as having high likelihood of exposure if the father reported an occupation in agriculture 14](#_Toc48063707)

[S12: Additional analysis comparing children for whom at least one parent had high likelihood of exposure to children whose parents both had no or minimal exposure 15](#_Toc48063708)

[S13: Change of parental occupational pesticides exposure between 1990 and 2000 16](#_Toc48063709)

### S1: Job categories assigned a high likelihood of exposure to pesticides by CLIC-JEM and their prevalence among parents of children included in the study.

| **Paternal exposure** | | | |
| --- | --- | --- | --- |
| **ISCO-88 code** | **Title of the job category** | **n** | **%** |
| 161 | Gardener (Military position)^1^ | 0 | 0 |
| 6110 | Market gardeners and crop growers heading^1^ | 0 | 0 |
| 6111 | Field crop and vegetable growers | 3,860 | 3.4 |
| 6112 | Tree and shrub crop growers | 453 | 0.4 |
| 6113 | Gardeners, horticultural and nursery growers | 13,226 | 11.6 |
| 6114 | Mixed-crop growers^1^ | 0 | 0 |
| 6121 | Dairy and livestock producers | 592 | 0.5 |
| 6130 | Market-oriented crop and animal producers | 91,210 | 80.2 |
| 7423 | Woodworking-machine setters and setters-operators | 2,095 | 1.8 |
| 9211 | Farm-hands and laborers | 2,348 | 2.1 |
| **Maternal exposure** | | | |
| **ISCO-88 code** | **Title of the job category** | **n** | **%** |
| 161 | Gardener (Military position)^1^ | 0 | 0 |
| 6110 | Market gardeners and crop growers heading^1^ | 0 | 0 |
| 6111 | Field crop and vegetable growers | 776 | 1.5 |
| 6112 | Tree and shrub crop growers | 111 | 0.2 |
| 6113 | Gardeners, horticultural and nursery growers | 6,798 | 12.8 |
| 6114 | Mixed-crop growers^1^ | 0 | 0 |
| 6121 | Dairy and livestock producers | 60 | 0.1 |
| 6130 | Market-oriented crop and animal producers | 43,395 | 81.8 |
| 7423 | Woodworking-machine setters and setters-operators | 54 | 0.1 |
| 9211 | Farm-hands and laborers | 1,880 | 3.5 |

^1^ No parents in the study population belonged to these categories of the job exposure matrix

### S2: Directed Acyclic Graphs (DAG) of known and suspected associations with childhood cancers (Potential confounders for which data were available and which were considered in the analyses are shown in bold)

Residential exposures:

- **Background ionizing radiation**
- **Degree of urbanization**
- **Air pollution (benzene exposure)**
- Agricultural pesticides
- **Swiss Neighborhood Socio-economic position**

Location of residence

Parental age at child’s birth

- **Maternal age**

Parental education

**Cancers in offspring (Outcome of interest)**

Parental occupation

Infections

Socio-economic position of the household

- **Education of the household reference person**

Birth weight

Parental occupational exposure to chemicals

- **Pesticides exposure (Exposure of interest)**
- **Benzene exposure**

Known

Hypothesized

### S3: Exposure prevalence among parents of children included in the analysis based on CLIC-JEM and reported job categories at censuses 1990 and 2000

| **Maternal exposure** | **Census 1990** | |  | **Census 2000** | |
| --- | --- | --- | --- | --- | --- |
| High likelihood (≥70% of people exposed) | 58,970 | *3.7* |  | 9,349 | *1.5* |
| Moderate likelihood (≥25% to <70%) | 1,077 | *0.1* |  | 449 | *0.1* |
| Limited likelihood (≥10 to <25%) | 27,726 | *1.8* |  | 11,004 | *1.7* |
| No or minimal likelihood (<10%) | 1,497,624 | *94.5* |  | 625,412 | *96.8* |
| Total | 1,585,397 | *100.0* |  | 646,214 | *100.0* |
| **Paternal exposure** |  | |  |  | |
| High likelihood (≥70% of people exposed) | 108,277 | *7.1* |  | 34,235 | *5.8* |
| Moderate likelihood (≥25% to <70%) | 28,524 | *1.9* |  | 10,562 | *1.8* |
| Limited likelihood (≥10 to <25%) | 87,951 | *5.8* |  | 40,637 | *6.9* |
| No or minimal likelihood (<10%) | 1,296,787 | *85.2* |  | 505,322 | *85.5* |
| Total | 1,521,539 | *100.0* |  | 590,756 | *100.0* |

### S4: Frequency of paternal and maternal occupational exposure to pesticides among children included in both analyses (n= 1,407,503)

|  | Fathers | |
| --- | --- | --- |
| Mothers | Minimal likelihood | High likelihood |
| Minimal likelihood | 1,304,288 | 53,272 |
| High likelihood | 6,397 | 43,546 |

### S5: Association between potential confounders and likelihood of paternal exposure to pesticides

| **Levels of exposure** | **Minimal** | | **Limited** | | **Moderate** | | **High** | | **P^4^** |
| --- | --- | --- | --- | --- | --- | --- | --- | --- | --- |
| **Potential confounders** | **n** | *%* | **n** | *%* | **n** | *%* | **n** | *%* |  |
| **Total** | 1451344 | *100* | 104304 | *100* | 30717 | *100.0* | 113784 | *100* |  |
| **Maternal age at birth** |  |  |  |  |  |  |  |  |  |
| <25 | 309887 | *21.4* | 29327 | *28.1* | 8670 | *28.2* | 28366 | *24.9* | <0.001 |
| 25-29 | 445325 | *30.7* | 34455 | *33* | 10571 | *34.4* | 37769 | *33.2* |  |
| 30-34 | 359793 | *24.8* | 21078 | *20.2* | 6119 | *19.9* | 23706 | *20.8* |  |
| ≥35 | 147672 | *10.2* | 7812 | *7.5* | 2014 | *6. 6* | 11025 | *9.7* |  |
| missing | 188667 | *13* | 11632 | *11.2* | 3343 | *10.9* | 12918 | *11.4* |  |
| **Education of ref person household** |  |  |  |  |  |  |  |  |  |
| Compulsory education or less | 222255 | *15.3* | 22887 | *21.9* | 4217 | *13.7* | 26389 | *23.2* | <0.001 |
| Upper secondary level education | 723584 | *49.9* | 67436 | *64.7* | 18070 | *58.8* | 64666 | *56.8* |  |
| Tertiary level education | 495115 | *34.1* | 12832 | *12.3* | 8270 | *26.9* | 22160 | *19.5* |  |
| Not known | 10390 | *0.7* | 1149 | *1.1* | 160 | *0.52* | 569 | *0.5* |  |
| **Swiss-SEP^1^** |  |  |  |  |  |  |  |  |  |
| Q1 | 299493 | *20.2* | 30239 | *29* | 9012 | *29.3* | 45336 | *39.8* | <0.001 |
| Q2 | 264485 | *18.2* | 22817 | *21.9* | 7045 | *22.9* | 24525 | *21.6* |  |
| Q3 | 260420 | *17.9* | 18551 | *17.8* | 5776 | *18.8* | 16465 | *14.5* |  |
| Q4 | 263511 | *18.2* | 14685 | *14.1* | 4110 | *13.4* | 10329 | *9.1* |  |
| Q5 | 235920 | *16.3* | 9013 | *8.6* | 2042 | *6.7* | 4760 | *4.2* |  |
| missing | 127515 | *8.8* | 8999 | *8.6* | 2732 | *8.9* | 12369 | *10.9* |  |
| **Degree of urbanization** |  |  |  |  |  |  |  |  |  |
| Urban | 342326 | *23.6* | 23327 | *22.4* | 3790 | *12.3* | 6023 | *5.3* | <0.001 |
| Semi-urban | 702162 | *48.4* | 42518 | *40.8* | 10574 | *34.4* | 29420 | *25.9* |  |
| Rural | 406856 | *28.0* | 38459 | *36.9* | 16353 | *53.2* | 78341 | *68.9* |  |
| **Paternal occupational exposure to benzene^2^** |  |  |  |  |  |  |  |  |  |
| 0 | 1214985 | *83.7* | 81004 | *77.7* | 17244 | *56.1* | 113784 | *100.0* | <0.001 |
| > 0-2 | 158877 | *11.0* | 21078 | *20.2* | 13356 | *43.5* | 0 | *0.0* |  |
| > 2-10 | 23450 | *1.6* | 2222 | *2.1* | 117 | *0.4* | 0 | *0.0* |  |
| > 10 | 54032 | *3.7* | 0 | *0* | 0 | *0.0* | 0 | *0.0* |  |
| **Dose total NBR (nSv/h)^3^** |  |  |  |  |  |  |  |  |  |
| <100 nSv/h | 552494 | *38.1* | 39076 | *37.5* | 12368 | *40.3* | 50441 | *44.3* | <0.001 |
| 100-150 | 787992 | *54.3* | 54943 | *52.7* | 15849 | *51.6* | 56262 | *49.4* |  |
| 150-200 | 94008 | *6.5* | 8639 | *8.3* | 2079 | *6.8* | 5205 | *4.6* |  |
| ≥250 | 2413 | *0.2* | 326 | *0.3* | 5205 | *0.3* | 245 | *0.2* |  |
| missing | 14437 | *1.0* | 1320 | *1.3* | 80 | *1.1* | 1631 | *1.4* |  |
| **Air pollution (NO_2_ pg/m^3^)** |  |  |  |  |  |  |  |  |  |
| Q1 | 328942 | *22.7* | 31545 | *30.2* | 12809 | *41.7* | 67084 | *59.0* | <0.001 |
| Q2 | 356604 | *24.6* | 24425 | *23.4* | 7267 | *23.7* | 27260 | *24.0* |  |
| Q3 | 370867 | *25.6* | 22671 | *21.7* | 5429 | *17.7* | 11174 | *9.8* |  |
| Q4 | 361093 | *24.9* | 23147 | *22.2* | 4253 | *13.9* | 5116 | *4.5* |  |
| Missing | 33838 | *2.3* | 2516 | *2.4* | 959 | *3.12* | 3150 | *2.8* |  |

Data represent number of children and column percentages *(in italic)*

^1^ Swiss-SEP is an area-based measure of socio-economic position for Switzerland, estimated in neighbourhoods of 50 households with a principal component analysis of four socio-economic variables using data from census 2000^26^

^2^Product of the proportion of workers exposed and the level of exposure [ppm] (at time of census) based on a job exposure matrix (BEN-JEM) and occupation at census^23^

^3^Estimated dose rate from terrestrial and cosmic background radiation based on a geographic exposure model^27^

^4^ p-value of the Chi-square test

### S6: Association between potential confounders and likelihood of maternal exposure to pesticides

| **Levels of exposure** | **Minimal** | | **Limited** | | **Moderate** | | **High** | | **P^4^** |
| --- | --- | --- | --- | --- | --- | --- | --- | --- | --- |
| **Potential confounders** | **n** | *%* | **n** | *%* | **n** | *%* | **n** | *%* |  |
| **Total** | 1721757 | *100.0* | 31838 | *100* | 1233 | *100* | 53074 | *100* |  |
| **Maternal age at birth** |  |  |  |  |  |  |  |  |  |
| <25 | 394982 | *22.9* | 10892 | *34.2* | 420 | *34.1* | 13367 | *25.2* | <0.001 |
| 25-29 | 532680 | *30.9* | 10172 | *32* | 387 | *31.4* | 17943 | *33.8* |  |
| 30-34 | 416788 | *24.2* | 5678 | *17.8* | 225 | *18.3* | 10963 | *20.7* |  |
| ≥35 | 176441 | *10.3* | 2185 | *6.9* | 95 | *7.7* | 5220 | *9.8* |  |
| missing | 170 | *11.7* | 2911 | *9.1* | 106 | *8.6* | 5581 | *10.5* |  |
| **Education of ref person household** |  |  |  |  |  |  |  |  |  |
| Compulsory education or less | 293592 | *17.1* | 9806 | *30.8* | 303 | *24.6* | 11394 | *21.5* | <0.001 |
| Upper secondary level education | 879571 | *51.1* | 17728 | *55.7* | 728 | *59.0* | 31204 | *58.8* |  |
| Tertiary level education | 529316 | *30.7* | 3834 | *12* | 190 | *15.4* | 10363 | *19.5* |  |
| Not known | 19278 | *1.1* | 470 | *1.5* | 12 | *1.0* | 113 | *0.2* |  |
| **Swiss-SEP^1^** |  |  |  |  |  |  |  |  |  |
| Q1 | 382828 | *22.2* | 9155 | *28.8* | 328 | *26.6* | 20059 | *37.8* | <0.001 |
| Q2 | 320330 | *18.6* | 6650 | *20.9* | 261 | *21.2* | 11160 | *21.0* |  |
| Q3 | 306302 | *17.8* | 5483 | *17.2* | 224 | *18.2* | 7714 | *14.5* |  |
| Q4 | 299430 | *17.4* | 4527 | *14.2* | 184 | *14.9* | 5168 | *9.7* |  |
| Q5 | 259430 | *15.1* | 2787 | *8.8* | 86 | *7.0* | 2431 | *4.6* |  |
| missing | 153454 | *8.9* | 3236 | *10.2* | 150 | *12.2* | 6542 | *12.3* |  |
| **Degree of urbanization** |  |  |  |  |  |  |  |  |  |
| Urban | 410271 | *23.8* | 9092 | *28.6* | 252 | *20.4* | 2517 | *4.7* | <0.001 |
| Semi-urban | 806997 | *46.9* | 13910 | *43.7* | 503 | *40.8* | 13370 | *25.2* |  |
| Rural | 504489 | *29.3* | 8836 | *27.8* | 478 | *38.8* | 37187 | *70.1* |  |
| **Maternal occupational exposure to benzene^2^** |  |  |  |  |  |  |  |  |  |
| 0 | 985030 | *57.2* | 31110 | *97.7* | 1169 | *94.8* | 53074 | *100.0* | <0.001 |
| > 0-2 | 61078 | *3.6* | 724 | *2.3* | 51 | *4.1* | 0 | *0.0* |  |
| > 2-10 | 4544 | *0.3* | 4 | *0.01* | 13 | *1.1* | 0 | *0.0* |  |
| > 10 | 671105 | *39.0* | 0 | *0* | 0 | *0* | 0 | *0.0* |  |
| **Dose total NBR (nSv/h)^3^** |  |  |  |  |  |  |  |  |  |
| <100 nSv/h | 652161 | *37.9* | 11104 | *34.9* | 489 | *39.7* | 24298 | *45.8* | <0.001 |
| 100-150 | 933312 | *54.2* | 17747 | *55.7* | 673 | *54.6* | 25993 | *49.0* |  |
| 150-200 | 114161 | *6.6* | 2544 | *8.0* | 56 | *4.5* | 2016 | *3.8* |  |
| ≥250 | 3098 | *0.2* | 43 | *0.1* | 3 | *0.2* | 104 | *0.2* |  |
| missing | 19022 | *1.1* | 400 | *1.3* | 12 | *1* | 663 | *1.2* |  |
| **Air pollution (NO_2_ pg/m^3^)** |  |  |  |  |  |  |  |  |  |
| Q1 | 413169 | *24* | 6608 | *20.8* | 358 | *29.0* | 29964 | *56.5* | <0.001 |
| Q2 | 421825 | *24.5* | 7229 | *22.7* | 351 | *28.5* | 14313 | *27.0* |  |
| Q3 | 428380 | *24.9* | 7558 | *23.7* | 269 | *21.8* | 5671 | *10.7* |  |
| Q4 | 417995 | *24.3* | 9975 | *31.3* | 228 | *18.5* | 2287 | *4.3* |  |
| Missing | 40388 | *2.4* | 468 | 1.5 | 27 | *2.2* | 839 | *1.6* |  |

Data represent number of children and column percentages *(in italic)*

^1^ Swiss-SEP is an area-based measure of socio-economic position for Switzerland, estimated in neighbourhoods of 50 households with a principal component analysis of four socio-economic variables using data from census 2000^26^

^2^Product of the proportion of workers exposed and the level of exposure [ppm] (at time of census) based on a job exposure matrix (BEN-JEM) and occupation at census^23^

^3^Estimated dose rate from terrestrial and cosmic background radiation based on a geographic exposure model^27^

^4^ p-value of the Chi-square test

### S7: Cases of childhood cancers included in analyses and likelihood of parental occupational exposure to pesticides

| **Exposure  Cancers** | **Total cases** | **High likelihood  (≥70% of people exposed)** | | **Moderate likelihood  (≥25% to <70%)** | | **Limited likelihood  (≥10 to <25%)** | | **No or minimal likelihood (<10%)** | |
| --- | --- | --- | --- | --- | --- | --- | --- | --- | --- |
|  |  | n | % | n | % | n | % | n | % |
| **Paternal exposure** |  |  |  |  |  |  |  |  |  |
| Any cancer | 1808 | 112 | ***6.2*** | 29 | ***1.6*** | 108 | ***6.0*** | 1559 | ***86.2*** |
| Leukaemia | 503 | 24 | ***4.8*** | 9 | ***1.8*** | 32 | ***6.4*** | 438 | ***87.1*** |
| LL | 383 | 19 | ***5.0*** | 7 | ***1.8*** | 25 | ***6.5*** | 332 | ***86.7*** |
| AML | 85 | 3 | ***3.5*** | 2 | ***2.4*** | 5 | ***5.9*** | 75 | ***88.2*** |
| Lymphoma | 337 | 21 | ***6.2*** | 6 | ***1.8*** | 13 | ***3.9*** | 297 | ***88.1*** |
| NHL | 169 | 8 | ***4.7*** | 4 | ***2.4*** | 6 | ***3.6*** | 151 | ***89.3*** |
| HL | 162 | 13 | ***8.0*** | 2 | ***1.2*** | 7 | ***4.3*** | 140 | ***86.4*** |
| CNST | 399 | 20 | ***3.5*** | 8 | ***1.4*** | 26 | ***4.6*** | 345 | ***60.6*** |
| Non-CNS solid tumours | 569 | 47 | ***8.3*** | 6 | ***1.1*** | 37 | ***6.5*** | 479 | ***84.2*** |
| **Maternal exposure** |  |  |  |  |  |  |  |  |  |
| Any cancer | 1891 | 49 | ***2.6*** | 1 | ***0.1*** | 33 | ***1.7*** | 1808 | ***95.6*** |
| Leukaemia | 532 | 9 | ***1.7*** | 1 | ***0.2*** | 7 | ***1.3*** | 515 | ***96.8*** |
| LL | 409 | 8 | ***2.0*** | 1 | ***0.2*** | 3 | ***0.7*** | 397 | ***97.1*** |
| AML | 89 | 1 | ***1.1*** | 0 | ***0.0*** | 4 | ***4.5*** | 84 | ***94.4*** |
| Lymphoma | 348 | 9 | ***2.6*** | 0 | ***0.0*** | 6 | ***1.7*** | 333 | ***95.7*** |
| NHL | 172 | 4 | ***2.3*** | 0 | ***0.0*** | 2 | ***1.2*** | 166 | ***96.5*** |
| HL | 170 | 4 | ***2.4*** | 0 | ***0.0*** | 4 | ***2.4*** | 162 | ***95.3*** |
| CNST | 423 | 8 | ***1.9*** | 0 | ***0.0*** | 10 | ***2.4*** | 405 | ***95.7*** |
| Non-CNS solid tumours | 588 | 23 | ***3.9*** | 0 | ***0.0*** | 10 | ***1.7*** | 555 | ***94.4*** |

LL: lymphoid leukaemia; AML: acute myeloid leukaemia; NHL: Non-Hodgkin’s lymphoma; HL: Hodgkin’s lymphoma; CNST: central nervous system tumour

### S8: Association between parental occupational exposure to pesticides and risk of childhood cancer in the Swiss National Cohort

|  |  | | **Paternal exposure** | | | |  | | **Maternal exposure** | | |
| --- | --- | --- | --- | --- | --- | --- | --- | --- | --- | --- | --- |
|  | | Likelihood of  Exposure | | Cases | Partially adjusted model^1^ | Fully adjusted model^2^ | |  | Cases | Partially adjusted model^1^ | Fully adjusted model^2^ |
|  | |  | | n | HR [95%CI] | HR [95%CI] | |  | n | HR [95%CI] | HR [95%CI] |
| Any cancer | | Minimal | | 1559 | 1.00 | 1.00 | |  | 1808 | 1.00 | 1.00 |
|  | | Limited | | 108 | 0.94[0.77-1.14] | 1.00[0.81-1.25] | |  | 33 | 1.08[0.76-1.52] | 1.11[0.76-1.62] |
|  | | Moderate | | 29 | 0.83[0.58-1.21] | 0.90[0.59-1.37] | |  | 1 | - | - |
|  | | High | | 112 | 0.95[0.79-1.16] | 1.14[0.91-1.43] | |  | 49 | 1.00[0.75-1.33] | 1.13[0.82-1.56] |
| Leukaemia | | Minimal | | 438 | 1.00 | 1.00 | |  | 515 | 1.00 | 1.00 |
|  | | Limited | | 32 | 0.99[0.69-1.42] | 1.04[0.70-1.55] | |  | 7 | 0.81[0.38-1.70] | 0.88[0.39-1.98] |
|  | | Moderate | | 9 | 0.92[0.47-1.78] | 1.11[0.55-2.26] | |  | 1 | - | - |
|  | | High | | 24 | 0.73[0.49-1.10] | 0.79[0.48-1.29] | |  | 9 | 0.66[0.34-1.27] | 0.66[0.29-1.49] |
| Lymphoma | | Minimal | | 297 | 1.00 | 1.00 | |  | 333 | 1.00 | 1.00 |
|  | | Limited | | 13 | 0.60[0.34-1.04] | 0.56[0.29-1.05] | |  | 6 | 1.05[0.47-2.35] | 1.01 [0.41-2.47] |
|  | | Moderate | | 6 | 0.92[0.41-2.05] | 0.77[0.28-2.08] | |  | 0 | - | - |
|  | | High | | 21 | 0.92[0.59-1.44] | 1.06[0.63-1.78] | |  | 9 | 0.96[0.49-1.86] | 1.18[0.57-2.44] |
| CNST | | Minimal | | 345 | 1.00 | 1.00 | |  | 405 | 1.00 | 1.00 |
|  | | Limited | | 26 | 1.01[0.68-1.51] | 1.34[0.88-2.06] | |  | 10 | 1.46[0.78-2.74] | 1.67 [0.85-3.28] |
|  | | Moderate | | 8 | 1.04[0.52-2.10] | 0.12[0.49-2.53] | |  | 0 | - | - |
|  | | High | | 20 | 0.78[0.50-1.22] | 0.76[0.44-1.34] | |  | 8 | 0.77[0.38-1.55] | 0.65[0.26-1.60] |
| Non-CNS solid tumours | | Minimal | | 479 | 1.00 | 1.00 | |  | 555 | 1.00 | 1.00 |
|  | | Limited | | 37 | 1.05[0.75-1.47] | 1.03[0.70-1.52] | |  | 10 | 1.06[0.57-1.99] | 0.99 [0.49-2.01] |
|  | | Moderate | | 6 | 0.56[0.25-1.25] | 0.65[0.26-1.57] | |  | 0 | - | - |
|  | | High | | 47 | 1.30[0.96-1.75] | 1.84[1.31-2.58] | |  | 23 | 1.49[0.98-2.26] | 1.79[1.13-2.84] |

CNST: central nervous system tumour; HR: Hazard Ratio estimated with a Cox regression; 95%CI: 95% confidence interval

^1^ Model adjusted for sex, birth year and census year at entry

^2^ Model adjusted for sex, birth year, maternal age at birth, census year at entry, paternal and maternal occupational exposure to benzene, education level of the reference person in the household, SEP-index, degree of urbanization, residential exposure to background ionising radiation, residential exposure to ambient NO_2_(All variables assessed at entry into the cohort)

### S9: Association between parental occupational exposure to pesticides and risk of childhood cancer in the Swiss National Cohort stratified by census year of entry (only outcomes with evidence of interaction shown)

|  |  |  | **Paternal exposure** | | | **Maternal exposure** | | |
| --- | --- | --- | --- | --- | --- | --- | --- | --- |
|  | Census year | Likelihood of exposure | Cases | Partially adjusted model^1^ | P^2^ | Cases | Partially adjusted model^1^ | P^2^ |
|  |  |  | n | HR [95%CI] |  | n | HR [95%CI] |  |
| Any cancer | 1990 | Minimal | 870 | 1 | 0.02 | 976 | 1 | 0.04 |
|  |  | High | 55 | 0.77[0.59-1.01] |  | 30 | 0.81[0.57-1.17] |  |
|  | 2000 | Minimal | 689 | 1 |  | 832 | 1 |  |
|  |  | High | 57 | 1.23[0.94-1.62] |  | 19 | 1.62[1.00-2.62] |  |
| Lymphoma | 1990 | Minimal | 174 | 1 | 0.03 |  |  |  |
|  |  | High | 8 | 0.55[0.27-1.12] |  |  |  |  |
|  | 2000 | Minimal | 123 | 1 |  |  |  |  |
|  |  | High | 13 | 1.54[0.87-2.73] |  |  |  |  |

HR: Hazard Ratio estimated with a Cox regression; 95%CI: 95% confidence interval

^1^ Model adjusted for sex, birth year

^2^ P-value of the log-likelihood test comparing the model with interaction between census year at entry and pesticides exposure and the model without interaction

### S10: Association between parental occupational exposure to pesticides and risk of childhood cancer in the Swiss National Cohort, classifying non-economically active parents as missing

|  |  | | **Paternal exposure** | | | |  | | **Maternal exposure** | | |
| --- | --- | --- | --- | --- | --- | --- | --- | --- | --- | --- | --- |
|  | | Likelihood of  Exposure | | Cases | Partially adjusted model^1^ | Fully adjusted model^2^ | |  | Cases | Partially adjusted model^1^ | Fully adjusted model^2^ |
|  | |  | | n | HR [95%CI] | HR [95%CI] | |  | n | HR [95%CI] | HR [95%CI] |
| Any cancer | | Minimal | | 1518 | 1 | 1 | |  | 1088 | 1 | 1 |
|  | | High | | 112 | 0.95[0.79-1.16] | 1.15[0.92-1.44] | |  | 49 | 0.98[0.73-1.30] | 1.14[0.82-1.59] |
| Leukaemia | | Minimal | | 428 | 1 | 1 | |  | 299 | 1 | 1 |
|  | | High | | 24 | 0.73[0.48-1.10] | 0.79[0.48-1.30] | |  | 9 | 0.66[0.34-1.28] | 0.64[0.28-1.47] |
| Lymphoma | | Minimal | | 291 | 1 | 1 | |  | 201 | 1 | 1 |
|  | | High | | 21 | 0.92[0.59-1.43] | 1.08[0.64-1.81] | |  | 9 | 0.94[0.48-1.84] | 1.21[0.58-2.52] |
| CNST | | Minimal | | 336 | 1 | 1 | |  | 241 | 1 | 1 |
|  | | High | | 20 | 0.78[0.50-1.22] | 0.77[0.44-1.34] | |  | 8 | 0.74[0.37-1.50] | 0.64[0.26-1.60] |
| Non-CNS solid tumours | | Minimal | | 463 | 1 | 1 | |  | 347 | 1 | 1 |
|  | | High | | 47 | 1.31[0.97-1.77] | 1.84[1.31-2.58] | |  | 23 | 1.41[0.93-2.16] | 1.88[1.18-3.00] |

CNST: central nervous system tumour; HR: Hazard Ratio estimated with a Cox regression; 95%CI: 95% confidence interval

^1^ Model adjusted for sex, birth year and census year at entry

^2^ Model adjusted for sex, birth year, maternal age at birth, census year at entry, paternal and maternal occupational exposure to benzene, education level of the reference person in the household, SEP-index, degree of urbanization, residential exposure to background ionizing radiation, residential exposure to ambient NO_2_(All variables assessed at entry into the cohort)

### S11: Sensitivity analysis classifying mothers reporting “housework” as having high likelihood of exposure if the father reported an occupation in agriculture

|  |  | **Maternal exposure** | | |
| --- | --- | --- | --- | --- |
| Outcome | Likelihood of  Exposure | Cases | Partially adjusted model^1^ | Fully adjusted model^2^ |
|  |  | n | HR [95%CI] | HR [95%CI] |

| Any cancer | Minimal | 1777 | 1 | 1 |
| --- | --- | --- | --- | --- |
|  | High | 80 | 0.89[0.71-1.12] | 1.05[0.81-1.35] |
| Leukaemia | Minimal | 508 | 1 | 1 |
|  | High | 16 | 0.63[0.38-1.04] | 0.61[0.33-1.13] |
| Lymphoma | Minimal | 329 | 1 | 1 |
|  | High | 13 | 0.76[0.44-1.33] | 0.87[0.47-1.62] |
| CNST | Minimal | 402 | 1 | 1 |
|  | High | 11 | 0.56[0.31-1.03] | 0.58[0.28-1.19] |
| Non-CNS solid tumours | Minimal | 538 | 1 | 1 |
|  | High | 40 | 1.44[1.04-1.99] | 1.90[1.33-2.72] |

CNST: central nervous system tumour; HR: Hazard Ratio estimated with a Cox regression; 95%CI: 95% confidence interval

^1^ Model adjusted for sex, birth year and census year at entry

^2^ Model adjusted for sex, birth year, maternal age at birth, census year at entry, paternal and maternal occupational exposure to benzene, education level of the reference person in the household, SEP-index, degree of urbanization, residential exposure to background ionizing radiation, residential exposure to ambient NO_2_(All variables assessed at entry into the cohort)

### S12: Additional analysis comparing children for whom at least one parent had high likelihood of exposure to children whose parents both had no or minimal exposure

|  | Likelihood of  Exposure | Cases | Partially adjusted model^1^ | Fully adjusted model^2^ |
| --- | --- | --- | --- | --- |
|  |  | n | HR [95%CI] | HR [95%CI] |
| Any cancer | Minimal both parents | 1390 | 1 | 1 |
|  | At least one high | 121 | 0.95[0.79-1.15] | 1.15[0.92-1.43] |
| Leukaemia | Minimal both parents | 385 | 1 | 1 |
|  | At least one high | 26 | 0.74[0.50-1.10] | 0.85[0.53-1.37] |
| Lymphoma | Minimal both parents | 264 | 1 | 1 |
|  | At least one high | 23 | 0.94[0.61-1.44] | 1.02[0.61-1.68] |
| CNST | Minimal both parents | 311 | 1 | 1 |
|  | At least one high | 20 | 0.71[0.45-1.11] | 0.72[0.41-1.27] |
| Non-CNS solid | Minimal both parents | 430 | 1 | 1 |
| tumours | At least one high | 52 | 1.32[0.99-1.76] | 1.80[1.30-2.51] |

CNST: central nervous system tumour; HR: Hazard Ratio estimated with a Cox regression; 95%CI: 95% confidence interval

^1^ Model adjusted for sex, birth year and census year at entry

^2^ Model adjusted for sex, birth year, maternal age at birth, census year at entry, paternal and maternal occupational exposure to benzene, education level of the reference person in the household, SEP-index, degree of urbanization, residential exposure to background ionizing radiation, residential exposure to ambient NO_2_(All variables assessed at entry into the cohort

### S13: Change of parental occupational pesticides exposure between 1990 and 2000

| 1) Exposure status in 2000 of parents with high likelihood in 1990 | | | |  |
| --- | --- | --- | --- | --- |
|  | Fathers | | Mothers | |
| Exposure status | N | % | N | % |
| High likelihood | 36,430 | 90.4 | 8,170 | 70.4 |
| Moderate | 308 | 0.8 | 34 | 0.3 |
| Limited | 510 | 1.3 | 190 | 1.6 |
| Minimal | 3,038 | 7.5 | 3,210 | 27.7 |
| Total | 40,286 | 100 | 11,604 | 100 |
| 2) Exposure status in 2000 of parents with minimal likelihood 1990 | | | |  |
|  | Fathers | | Mothers | |
| Exposure status | N | % | N | % |
| High likelihood | 3,368 | 0.9 | 5,152 | 1.5 |
| Moderate | 1,532 | 0.4 | 376 | 0.1 |
| Limited | 10,744 | 2.8 | 10,334 | 3.1 |
| Minimal | 365,432 | 95.9 | 319,478 | 95.3 |
| Total | 381,076 | 100 | 335,340 | 100 |
